# Breaking the cycle: a review of the impacts of maternal PTSD, adverse pregnancy outcomes, and health disparities among Black women

**DOI:** 10.1186/s40748-026-00289-0

**Published:** 2026-08-17

**Authors:** Tiffany R. Williams, Christina Scifres, David M. Haas

**Affiliations:** 1 Department of Psychiatry, Indiana University School of Medicine, 355 West 16th St., Indianapolis, IN 46202 USA; 2 Department of Obstetrics and Gynecology, Indiana University School of Medicine, Indianapolis, IN USA

**Keywords:** Maternal PTSD, Adverse pregnancy outcomes, Maternal morbidity, NICU, Trauma-informed care, Health disparities, Black maternal health, Policy reform, Economic burden, Culturally responsive interventions

## Abstract

Posttraumatic stress disorder (PTSD) is an underrecognized contributor to maternal morbidity, infant health complications, and rising healthcare costs. This narrative review synthesizes interdisciplinary evidence on the pathways linking structural inequities, trauma exposure, and maternal PTSD to adverse maternal and infant outcomes, with particular attention to the disproportionate burden experienced by Black women. Structural racism and systemic inequities increase exposure to chronic stress and trauma, which in turn elevate risk for PTSD during the perinatal period. Maternal PTSD functions as a central mechanism linking upstream inequities to downstream health outcomes. Through dysregulation of stress-responsive biological systems and associated behavioral pathways, PTSD increases the risk of adverse maternal outcomes, including hypertensive disorders, preterm birth, and severe maternal morbidity, as well as infant outcomes such as low birth weight, neurodevelopmental and emotional regulation difficulties, and impaired mother–infant attachment. These relationships are bidirectional, with adverse pregnancy and birth experiences further contributing to the onset or exacerbation of PTSD symptoms, reinforcing a cycle of psychological and physiological vulnerability. These interconnected maternal and infant outcomes contribute to substantial healthcare utilization and economic burden at individual and societal levels. The cumulative impact of perinatal mental health conditions exceeds $14 billion annually in the United States, driven in part by adverse birth outcomes and downstream developmental consequences. Addressing this cycle requires trauma-informed, integrated care models that incorporate universal screening, improve access to evidence-based treatment, and address structural barriers to care. Developing scalable, culturally responsive interventions may help reduce disparities, improve maternal and infant outcomes, and mitigate long-term societal costs.

## Introduction

Mental illness is a leading contributor to pregnancy-related deaths in the United States (U.S.) [1, 2]. Although overall maternal mortality rates have declined for White, Hispanic, and Asian women, they increased alarmingly for Black women in 2023, reaching 50.3 deaths per 100,000 live births [2, 3]. Black women account for 31% of maternal deaths, [4, 5] despite comprising only 15% of the U.S. female population. Black women also experience disproportionately high rates of perinatal mental health symptoms, with nearly 40% reporting symptoms during the perinatal or postpartum period [6]. They experience significantly higher rates of mental illness during pregnancy than white women, [7–9] including disproportionate comorbid conditions, such as depression or anxiety [8, 10] and suicide risk [11–13]. Among the most urgent and overlooked threats to Black maternal health is posttraumatic stress disorder (PTSD) [14]. Individuals with trauma histories face substantially elevated mortality risk during the perinatal period, with Black women experiencing the highest rates of death [15]. Prioritizing Black maternal mental health, especially treatment for PTSD, is essential to improving pregnancy outcomes and reducing mortality and morbidity [16, 17].

In addition to vulnerability to PTSD, adverse pregnancy outcomes represent another major and preventable contributor to maternal and infant mortality among Black women. These outcomes, such as preeclampsia, preterm birth, and low birth weight, are among the leading causes of pregnancy- and infant-related deaths [4, 14]. Black women not only face these complications more frequently, but are also more likely to endure traumatic births as a result of these adverse outcomes, further compounding their risk [10, 18]. As a result, Black women are disproportionately affected by both PTSD and adverse pregnancy outcomes, often simultaneously. PTSD not only increases the risk of adverse pregnancy outcomes [19–23] but these complications can, in turn, trigger or exacerbate PTSD symptoms, [24] creating a reciprocal pattern of trauma and physiological vulnerability. Breaking this cycle requires targeted attention to the development and implementation of evidence-based treatments for maternal PTSD, particularly for Black women who face concurrent mental health and pregnancy-related risks.

Although some psychoeducational interventions have emerged, [25, 26] these primarily target mood and anxiety disorders rather than maternal PTSD. There is a growing need for brief, evidence-based PTSD intervention that can address barriers related to limited access and time constraints among perinatal individuals [27–30]. Traditional in-person therapies often exacerbate these challenges, restricting timely engagement with mental health care. Early screening and access to evidence-based trauma focused care remain limited, largely due to persistent racial and health disparities that marginalize Black perinatal women [16, 31]. Moreover, gold-standard PTSD treatments, such as cognitive behavioral therapy, cognitive processing therapy, and exposure-based approaches, have historically excluded perinatal populations altogether [34, 35].

There is a critical need for scalable, culturally responsive interventions to reduce symptoms of maternal PTSD among Black women, improve maternal and health outcomes, and alleviate the growing burden on healthcare systems [36, 37]. This narrative review highlights the need to develop and rigorously test interventions specifically tailored for Black women, one of the most vulnerable and underserved populations with perinatal and postpartum PTSD. Short-term, accessible treatment options, including virtual care are essential not only to reduce PTSD symptoms but also to improve engagement and equity in care. Without immediate action, the nation faces profound consequences: long-term developmental impacts on infants and escalating healthcare costs that strain families and systems alike.

## Review approach

A narrative review methodology was intentionally selected because the literature relevant to maternal PTSD spans multiple interconnected domains. These domains include structural and systemic inequities, cumulative trauma and stress exposure, the development of perinatal PTSD, downstream maternal and infant outcomes, and associated economic burden. These interconnected domains are not easily addressed within a single systematic or scoping framework. While systematic and scoping reviews are well suited for narrowly defined questions or cataloging intervention evidence, the present review aims to conceptually integrate clinical, psychosocial, and structural factors to elucidate the bidirectional cycle linking maternal PTSD and adverse pregnancy outcomes among Black women.

This narrative review synthesizes evidence across clinical, epidemiological, psychological, and public health literatures to examine the relationships among maternal PTSD, adverse pregnancy outcomes, and health disparities affecting Black women. Literature was identified through targeted searches of peer-reviewed journals and public health reports using key terms related to *maternal PTSD*, *perinatal trauma*, *adverse pregnancy outcomes*, *intimate partner violence*, *racial and structural health disparities*, and *Black maternal health*. Sources were selected to provide representation across clinical, epidemiological, psychological, public health, and policy literatures relevant to the conceptual framework of the review. Priority was given to systematic reviews, large observational studies, and recent high-quality empirical work, as well as seminal theoretical and policy papers relevant to trauma-informed and equity-centered care. Studies were selected to support conceptual integration across disciplines and to elucidate mechanisms and pathways, rather than to provide an exhaustive catalog of all available evidence. This review focuses on trauma exposure and PTSD in the context of pregnancy and the postpartum period, with an emphasis on pathways relevant to Black women in the U.S. It does not provide a comprehensive evaluation of intervention efficacy or a formal comparison of treatment outcomes, which remains an important area for future systematic and scoping reviews. Evidence linking maternal PTSD to adverse birth outcomes and maternal morbidity is relatively robust, whereas research on culturally responsive interventions and mechanisms of intergenerational transmission remains comparatively limited and preliminary.

### Structural and systemic inequities increase vulnerability

Structural and systemic oppression, including historical and gendered racism, profoundly shape Black women’s experiences of motherhood, reproductive decision-making, and healthcare access in the U.S. These intersecting forms of domination perpetuate subjugation, violence, and exclusion, as theorized by Black feminist and intersectionality scholars [38–40]. Such oppression manifests in gynecological and intergenerational trauma, which complicates pregnancy and birthing experiences and undermines the ability of Black women to have their needs met within healthcare systems. [41] Obstetric and gendered racism, along with broader inequities in sexual and reproductive healthcare, contribute to poor mental health outcomes during the perinatal and postpartum period [42–45].

These structural inequities not only shape exposure to adverse experiences but also contribute to trauma-related risk through specific psychosocial and biological pathways. Chronic exposure to racial discrimination, medical mistrust, and obstetric racism may increase vulnerability to posttraumatic stress by fostering heightened vigilance, perceived threat during healthcare encounters, and negative appraisal of pregnancy and childbirth experiences [43, 44, 46]. Additionally, prior trauma and ongoing stress exposure may compound risk for the development or exacerbation of PTSD during the perinatal period [47, 48].

These pathways extend beyond psychological risk to influence obstetric outcomes. Chronic stress and trauma-related dysregulation are associated with alterations in stress physiology, including neuroendocrine and inflammatory processes, which have been linked to adverse pregnancy outcomes such as preterm birth and low birth weight [21, 24]. Structural inequities may also contribute to adverse outcomes through reduced access to care, delayed treatment, and negative healthcare interactions, further increasing risk for maternal morbidity and mortality.

Together, these mechanisms highlight how structural inequities shape both risk for PTSD and adverse pregnancy outcomes through interconnected biological, psychosocial, and healthcare system pathways. Key drivers of these disparities include obstetric racism, racial discrimination, weathering, medical bias, and intimate partner violence, which operate across individual, interpersonal, and structural levels [42–44, 49]. Importantly, these inequities extend beyond childbirth, influencing postpartum recovery, infant health, and longer-term maternal outcomes. This framework underscores the need to understand disparities not only as background context, but as active mechanisms contributing to trauma exposure and perinatal health risk. Together, these structural inequities shape disproportionate exposure to trauma and chronic stress among Black women, contributing to elevated risk for PTSD during the perinatal period.

### Trauma and chronic stress exposure

Black women of childbearing age experience disproportionately high exposure to cumulative and severe trauma across the lifespan, including intimate partner violence, childhood sexual abuse, and community and interpersonal violence [48, 50–55]. Black women also face elevated risk of intimate partner homicide, further compounding trauma burden and risk for adverse mental health outcomes [51, 52]. These traumatic experiences often precede pregnancy; however, abuse during pregnancy also remains common [50, 51, 55]. Black women experience higher rates of physical abuse during pregnancy, with risk factors including being unmarried, receiving public assistance, living below the poverty line, smoking, and a history of preconception depression [53, 56]. In addition to these lifetime and ongoing exposures, Black women face markedly higher rates of traumatic childbirth, driven by systemic health disparities and an elevated likelihood of complicated pregnancies [22, 57, 58]. Consistent with these patterns, Black women are also at elevated risk for trauma-related disorders, including PTSD, acute stress disorder, and related conditions such as adjustment disorders with trauma-related features and subthreshold posttraumatic stress symptoms, both prior to and during the perinatal period [55, 59, 60].

Beyond discrete traumatic events, chronic exposure to psychosocial stressors may contribute to cumulative physiological wear and tear through processes commonly described as allostatic load [38, 43, 46, 61]. Persistent activation of physiological stress-response systems reflects cumulative biological wear and tear associated with chronic stress exposure. Repeated activation of stress-response systems resulting from chronic stress, discrimination, economic hardship, and other social adversities have been linked to adverse pregnancy outcomes, including hypertensive disorders of pregnancy and preterm birth. Repeated dysregulation of neuroendocrine, inflammatory, and other stress-responsive systems has been implicated in adverse pregnancy outcomes and may represent one pathway through which structural inequities become biologically embedded [62–65]. Recent findings from the Nulliparous Pregnancy Outcomes Study: Monitoring Mothers-to-Be (nuMoM2b) suggest that higher allostatic load is associated with increased risk of adverse pregnancy outcomes and may partially mediate racial disparities in these outcomes [66]. These findings provide additional support for understanding chronic stress as a biologically embedded pathway through which structural inequities influence maternal health risk across the perinatal period and may contribute to vulnerability for trauma-related mental health conditions, including PTSD.

### Maternal PTSD as a central mechanism

Cumulative exposure to trauma and chronic stress during the perinatal period positions posttraumatic stress disorder (PTSD) as a central mechanism linking upstream inequities and trauma exposures to downstream maternal and infant outcomes. The psychological burden of managing prior and ongoing trauma in the context of pregnancy may deplete emotional and cognitive resources, increasing the likelihood that pregnancy, labor, and delivery are experienced as distressing or traumatic events [38, 67, 68]. Although not all individuals exposed to traumatic birth experiences develop PTSD, clinically significant posttraumatic stress symptoms are common, with a subset meeting full diagnostic criteria [19, 21, 22]. Additional risk factors include perceived infant illness, prior obstetric complications, pre-existing psychiatric conditions, and inadequate support during neonatal intensive care unit (NICU) hospitalization [69].

PTSD rarely occurs in isolation. Comorbid conditions including depression and anxiety are highly prevalent among Black perinatal women and further contribute to adverse health risks [4, 7, 8, 37, 70]. These co-occurring conditions are associated with increased likelihood of pregnancy complications, maternal morbidity, and elevated risk of suicidality [11–13]. These comorbid mental health conditions, particularly depression and anxiety, are prevalent among Black perinatal women and are associated with adverse pregnancy outcomes. The prevalence of depression among Black women during the perinatal period is estimated at 10–15%, with higher rates among younger women aged 19–24 [4, 70]. Black women not only experience more severe perinatal depressive symptoms than white women [7], but also face nearly double the rates of postpartum depression [37]. Depressive symptoms and related mental health conditions have been linked to pregnancy complications, maternal morbidity, and increased risk of suicidality.

A primary pathway through which PTSD influences perinatal outcomes involves dysregulation of stress-responsive biological systems. Chronic activation of the hypothalamic–pituitary–adrenal (HPA) axis leads to sustained elevations in circulating glucocorticoids, including cortisol, which may disrupt placental functioning and fetal exposure to maternal stress signals [62, 63]. Altered placental glucocorticoid regulation has been linked to impaired fetal growth, shortened gestational duration, and increased risk of preterm birth [62, 63]. Prenatal stress exposure has also been associated with epigenetic modifications in stress-response genes (e.g., *NR3C1*, *FKBP5*), detectable at birth, suggesting a biologically plausible mechanism linking maternal PTSD to fetal development [64, 65].

Though these mechanisms are supported by converging epidemiological, translational, and molecular evidence, causal pathways in humans remain difficult to establish. Much of the mechanistic literature is correlational, and downstream processes including intergenerational transmission of stress vulnerability require further investigation. Nevertheless, these findings support a model in which PTSD acts as a central biological and psychological mechanism that shapes both maternal and infant health trajectories across the perinatal period.

### Maternal outcomes and the feedback loop

**Fig. 1:**
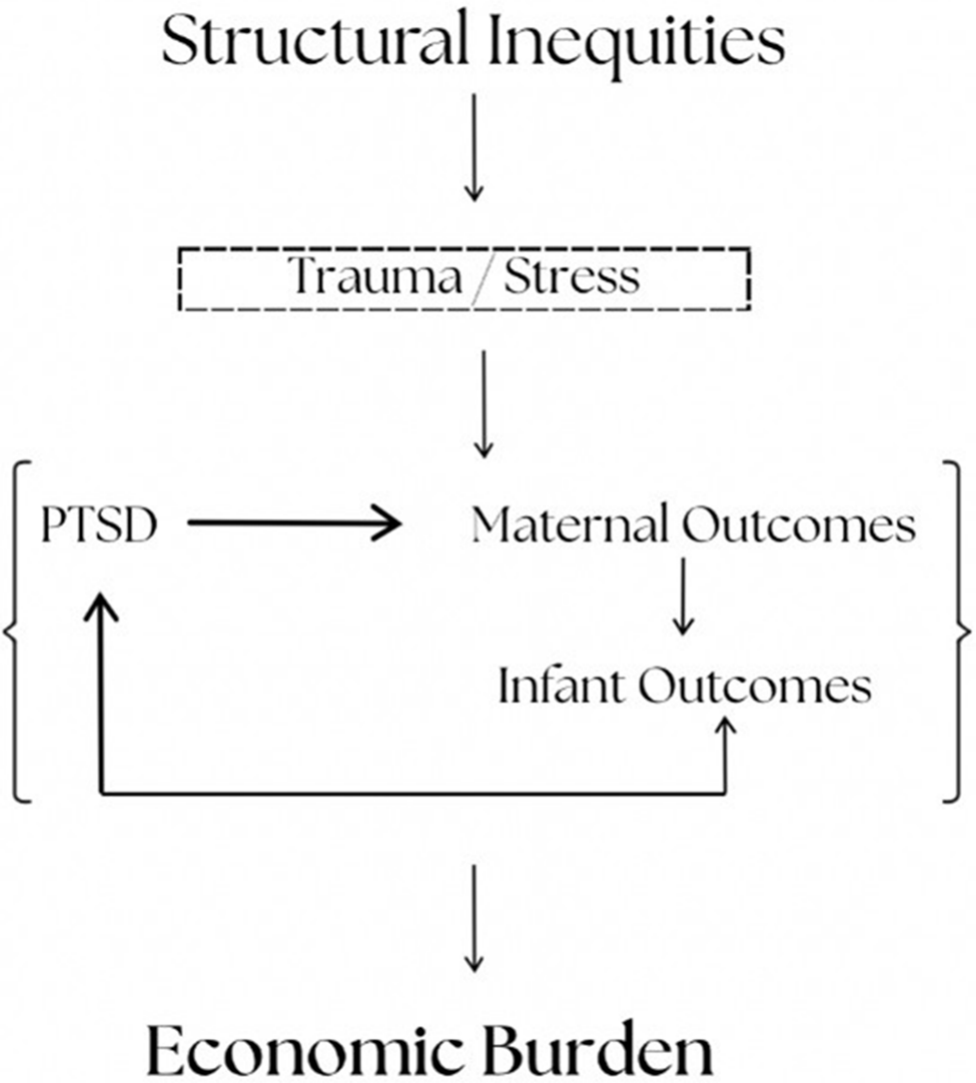
Conceptual model of the cyclical pathways linking maternal PTSD, physiological dysregulation, behavioral responses, and structural inequities in contributing to adverse maternal outcomes, reinforcing a feedback loop that sustains risk

Trauma-related physiological dysregulation contributes to adverse outcomes. PTSD is associated with chronic stress responses that can exacerbate conditions such as gestational diabetes and hypertension, compounding adverse outcomes [59]. In addition, behavioral and systemic factors including delayed prenatal care, medical mistrust, and barriers to healthcare access may amplify these risks within structurally inequitable systems [44, 46, 61, 71]. In addition, some adverse pregnancy outcomes may be indirectly influenced by coping behaviors associated with PTSD, including increased substance use, smoking, sleep disruption, and reduced engagement in prenatal care, which can further compound physiological and psychosocial risk during pregnancy, reinforcing a cyclical pathway through which PTSD and structural inequities sustain adverse maternal outcomes (see Fig. 1) [59, 72, 73].

Maternal PTSD contributes to a range of adverse pregnancy outcomes that significantly increase maternal morbidity and mortality risk. Black women experience a disproportionate burden of conditions including hypertensive disorders of pregnancy, cardiomyopathy, thrombotic events, hemorrhage, and mental health–related complications [4, 14]. Preterm birth and low birth weight are strongly associated with adverse maternal and infant outcomes and contribute to pregnancy-related morbidity and mortality [4, 14]. Among these, preeclampsia is particularly concerning, as it doubles the risk of peripartum cardiomyopathy and heart failure [31, 74]. Additionally, Black women are twice as likely to be diagnosed with Type 2 diabetes following gestational diabetes mellitus compared to white women, [49] while also facing elevated risks for acute ischemic heart disease, arrhythmias, and acute renal failure [49, 74].

Importantly, the relationship between maternal PTSD and adverse pregnancy outcomes is bidirectional. PTSD increases the likelihood of complications, while these complications can precipitate or exacerbate PTSD symptoms [18, 21, 22, 24]. For example, emergency deliveries, life-threatening obstetric events, or prolonged NICU admissions may reinforce perceptions of threat and loss of control, exacerbating PTSD symptom severity. This reciprocal relationship creates a feedback loop in which trauma-related symptoms and adverse health outcomes interact to produce cumulative maternal risk over time. Persistently elevated stress responses, repeated trauma exposure, and unresolved PTSD symptoms may contribute to worsening physical health, delayed recovery, and increased risk of future complications in subsequent pregnancies. Together, these findings underscore maternal PTSD as both a consequence and a driver of adverse maternal health outcomes within the perinatal period. Beyond the perinatal period, untreated or persistent maternal PTSD may contribute to longer-term impairments in psychological well-being, physical health, and overall functioning, with potential implications for chronic disease risk, relationship functioning, and sustained mental health burden across the lifespan [11–13].

### Infant outcomes

Maternal PTSD has significant downstream implications for infant health, extending from the perinatal period through early childhood and beyond. Infant outcomes associated with maternal PTSD arise through multiple, interacting pathways, including prenatal biological programming, perinatal health complications, and postnatal caregiving processes [75]. Beginning in the perinatal period, maternal PTSD is associated with increased risk of adverse birth and neonatal outcomes, including preterm birth, low birth weight, impaired fetal growth, and NICU admission [69, 73, 76]. These early health challenges are often accompanied by feeding difficulties, sleep disturbances, and physiological dysregulation. Chronic maternal stress exposure during pregnancy is thought to influence fetal development through neuroendocrine mechanisms, including sustained activation of the HPA axis, which may alter fetal stress-response systems [62, 63].

Beyond birth, infants exposed to maternal PTSD are at elevated risk for neurodevelopmental and behavioral difficulties. These include deficits in fine motor coordination, impaired adaptive functioning, and increased likelihood of both internalizing and externalizing symptoms across development [76, 77]. Prenatal stress exposure has also been associated with long-term alterations in emotional reactivity, heightened negative affectivity, and increased susceptibility to anxiety and depression [77, 78]. These outcomes are often conceptualized as reflecting prenatal programming of the infant’s stress-response system, resulting from exposure to elevated maternal stress hormones during gestation [79].

In addition to prenatal and biological pathways, maternal PTSD shapes infant outcomes through its impact on early caregiving relationships. Core PTSD symptoms including emotional numbing, avoidance, and hyperarousal, may interfere with maternal sensitivity, responsiveness, and attunement to infant cues [22, 78, 80]. Mothers with PTSD may exhibit reduced physical contact, disengagement during interactions, and difficulty accurately interpreting infant signals, leading to disruptions in dyadic synchrony [47, 77, 81, 82]. These disruptions may reflect impairments in maternal reflective functioning, reduced contingent responsiveness to infant cues, and difficulties regulating affect during caregiver–infant interactions [83]. Empirical studies suggest that maternal PTSD symptoms are associated with lower observed sensitivity and greater dyadic dysregulation in early infancy even after accounting for co-occurring mood and anxiety symptoms [48, 77, 78]. These relational challenges increase the risk of insecure attachment, which in turn has cascading effects on socioemotional development and later relational functioning [23, 75, 78]. Because early attachment relationships play a central role in the development of infant stress regulation and socioemotional functioning, these disruptions may alter developmental trajectories by shaping physiological reactivity, emotion regulation capacity, and later interpersonal functioning.

Taken together, these findings suggest that infant outcomes associated with maternal PTSD reflect a convergence of biological vulnerability, early-life health challenges, and disrupted caregiving environments. These interrelated processes highlight the potential for intergenerational transmission of trauma-related risk and underscore the importance of early identification and intervention to support both maternal mental health and infant developmental trajectories. These interconnected maternal and infant outcomes not only reflect significant clinical burden but also translate into substantial economic costs, reinforcing the broader societal impact of maternal PTSD across the perinatal period and early childhood.

### Economic burden

The clinical consequences of maternal PTSD are compounded by substantial economic costs at the individual, healthcare system, and societal levels. At the level of delivery hospitalization, perinatal mental health conditions are associated with approximately a 50% increased risk of severe maternal morbidity, contributing to higher per-delivery costs and an estimated $102 million in annual U.S. delivery-related expenditures [84]. Individuals with trauma- and stress-related disorders incur even greater burden, including higher costs per delivery and substantially elevated morbidity risk [84]. These findings suggest that trauma-related conditions, including PTSD, contribute disproportionately to acute healthcare utilization during the perinatal period.

At the population level, the economic burden becomes more pronounced when considering the cumulative impact of perinatal mental health conditions. Total societal costs are estimated at approximately $14 billion annually, reflecting maternal complications, psychiatric morbidity, and downstream infant outcomes [85, 86]. PTSD prevalence rivals that of other major obstetric complications and is consistently associated with severe outcomes, including suicidal ideation and maternal mortality during pregnancy and the postpartum period [11, 87, 88]. Together, these data underscore maternal PTSD as a high-risk, high-cost condition within perinatal care.

A substantial proportion of these costs reflects downstream expenditures related to infant and early childhood outcomes, highlighting the intergenerational economic impact of maternal mental health. Infant healthcare costs increase dramatically in the setting of adverse birth outcomes and neonatal complications. For example, while costs for infants without complications remain relatively modest, expenses associated with usual NICU admission are several-fold higher, particularly for extended stays [86, 89]. These costs are closely linked to conditions associated with maternal stress and trauma exposure, including preterm birth and low birth weight [24]. At a broader level, the economic burden of maternal morbidity across pregnancy and early childhood reaches tens of billions of dollars, with most costs attributable to child outcomes and concentrated in the early postnatal period [86, 90]. Importantly, these economic patterns reflect and may exacerbate persistent racial disparities in maternal and infant outcomes, with higher rates of complications and healthcare utilization among Black women and infants [31, 90]. Disproportionate rates of NICU admission and adverse birth outcomes contribute to inequitable cost distribution and reinforce structural inequities in perinatal health.

Maternal PTSD contributes to a bidirectional cycle of risk and cost, in which trauma-related symptoms increase the likelihood of adverse pregnancy and infant outcomes, while complications such as preterm birth or NICU admission further exacerbate PTSD symptoms. This feedback loop increases healthcare utilization, prolongs recovery, and amplifies both short- and long-term expenditures. Taken together, these findings position maternal PTSD as not only a significant clinical concern but also a key driver of intergenerational economic burden. Interventions that support early identification and access to culturally responsive, trauma-focused treatment may reduce healthcare utilization, prevent downstream complications, and mitigate long-term costs for both mothers and children [84, 91].

### Implications for clinical practice and health systems

Although multiple strategies have been proposed to address the intersection of maternal PTSD, adverse perinatal outcomes, and racial inequities, emerging evidence suggests that integrated obstetric–mental health care models and brief, scalable trauma-focused interventions are among the most promising and immediately actionable approaches within current health systems. These strategies align with existing clinical workflows and address persistent barriers to care, including workforce shortages and logistical constraints. Given rising rates of maternal morbidity and mortality in the U.S. particularly among Black women, identifying feasible, evidence-informed interventions that can be embedded within routine perinatal care is an urgent priority [1–3].

Integrated care models that embed mental health services within obstetric settings have demonstrated improvements in access, continuity, and quality of care, and are increasingly recognized as essential to addressing maternal mental health needs within routine care delivery [91, 92]. Within these models, routine trauma screening across preconception, prenatal, and postpartum periods serves as a critical entry point for identifying individuals at elevated risk, with systematic reviews supporting the effectiveness of screening programs for maternal mental health conditions [93]. However, screening alone is insufficient without timely access to evidence-based treatment, particularly interventions that can be delivered within time-limited clinical environments.

Among available interventions, brief, manualized trauma-focused therapies represent a particularly promising approach for perinatal settings. Exposure-based therapies are widely recognized as a gold standard for PTSD treatment, with strong evidence supporting their efficacy across populations [94]. Systematic reviews indicate that trauma-focused psychological therapies are effective in reducing postpartum PTSD symptoms, although research specific to perinatal populations remains limited relative to broader PTSD samples [95]. Narrative Exposure Therapy (NET) has been adapted for use in perinatal populations, with early feasibility and acceptability demonstrated in pregnant and postpartum samples [29, 96]. Written Exposure Therapy (WET), a brief intervention, has also shown feasibility in underserved Black perinatal populations and in obstetric clinical settings, suggesting promise for real-world implementation [97–99]. These interventions are particularly well-suited for perinatal care contexts because of their feasibility and scalability. Brief, structured protocols can be delivered within limited timeframes and integrated into high-risk obstetric settings, including inpatient units. They are also amenable to virtual delivery, which may reduce access barriers that disproportionately affect underserved populations [97, 98].

Despite this promise, implementation remains constrained by several persistent barriers. Integration efforts are frequently limited by reimbursement restrictions for behavioral health services, shortages of providers with perinatal mental health expertise, and structural barriers within healthcare systems that limit access to care for perinatal populations [91]. Broader system-level changes, including insurance expansion and workforce development, are critical but may not yield immediate impact without concurrent attention to delivery infrastructure. Health systems that prioritize coordinated care pathways, trauma-informed care, and culturally responsive practices are better positioned to mitigate these barriers and improve maternal and neonatal outcomes [36]. Together, these approaches support a pragmatic clinical pathway in which universal trauma screening is paired with immediate access to brief, scalable interventions embedded within integrated care models, with the potential to improve both maternal mental health and downstream infant outcomes, including birth outcomes and early relational development [21, 22, 24].

### System-level priorities and implementation considerations

While comprehensive reform is needed, prioritizing feasible, high-impact system-level strategies is essential. Three interrelated priorities emerge: (1) implementation of integrated care models, (2) expansion of access through scalable delivery approaches, and (3) development of structural supports that sustain implementation.

First, integrated care models represent a key foundation for improving perinatal mental health care. These models reduce fragmentation and facilitate timely intervention during periods of heightened vulnerability, including pregnancy complications and high-risk obstetric admissions. Evidence suggests that access to specialized psychiatric care, including inpatient and intensive perinatal mental health programs, can improve outcomes during acute care episodes [100, 101]. At the same time, ongoing calls to expand specialized perinatal mental health services highlight persistent gaps in availability and access [102]. Second, expanding access through scalable delivery approaches is critical. Brief interventions, inpatient-based care, and telehealth-enabled models can reduce common barriers such as transportation, childcare demands, and scheduling constraints. These approaches are particularly relevant for individuals experiencing medical complexity or prolonged hospitalization and may improve engagement in care among populations historically underserved by traditional outpatient models [98]. Third, structural supports are necessary to sustain implementation. Persistent barriers include limited insurance coverage, inadequate reimbursement for mental health services, and workforce shortages in perinatal psychiatry and psychology [91]. Addressing these challenges will require alignment between clinical practice, health system infrastructure, and payer policies. Given the substantial costs associated with untreated maternal mental health conditions including increased maternal morbidity and healthcare expenditures, investment in integrated, trauma-informed care models may yield downstream cost savings and improved health outcomes [84]. Importantly, emerging implementation efforts are beginning to evaluate these approaches in real-world settings, including adaptations of trauma-focused therapies within obstetric and inpatient contexts [98]. Continued research is needed to identify optimal strategies for scaling these models across diverse healthcare systems and populations.

### Limitations

Several limitations should be noted. As a narrative review, this work was not designed to provide a comprehensive or systematic assessment of all available evidence. Literature selection was intended to support conceptual integration across multiple disciplines rather than exhaustive coverage of individual research domains. Additionally, several areas discussed in this review, including biological mechanisms linking maternal PTSD to infant outcomes and culturally responsive intervention development, remain emerging areas of investigation. Findings should therefore be interpreted in light of the strengths and limitations of the underlying literature. Accordingly, conclusions regarding causal pathways and intervention effectiveness should be interpreted cautiously and areas for continued empirical investigation.

### Future directions for research

Future research should prioritize interdisciplinary and implementation-focused approaches that address both clinical efficacy and real-world feasibility. Fragmented and siloed approaches to maternal mental health continue to limit care delivery and perpetuate disparities in outcomes, underscoring the need for integrated, systems-level research frameworks [61, 91].

A key priority is the development and evaluation of scalable, culturally responsive PTSD interventions tailored for perinatal populations, particularly those that can be delivered in brief formats or through digital and hybrid care models. Existing work demonstrates the feasibility of trauma-focused interventions, including NET and WET, in perinatal samples and obstetric settings; however, larger-scale trials are needed to establish effectiveness across diverse populations and care contexts [29, 96–98]. Interventions that balance clinical effectiveness with feasibility, accessibility, and scalability will be essential for broader implementation.

In parallel, mechanistic research is needed to elucidate biological and psychosocial pathways linking maternal PTSD to adverse maternal and infant outcomes. Prior work has identified associations between maternal stress and dysregulation in neuroendocrine, inflammatory, and epigenetic processes, with downstream effects on fetal development and infant health [62–65, 103]. Further studies should examine how trauma-related symptom processes influence these pathways across the perinatal period and identify critical windows for intervention.

Additionally, research examining the role of structural racism, chronic stress exposure, and social determinants of health is essential for understanding how systemic inequities shape trauma exposure and contribute to maternal morbidity among Black women. Existing literature highlights the impact of racial discrimination, social disadvantage, and structural inequities on perinatal mental health and associated outcomes [44, 61, 71]. Future studies should integrate these contextual factors into intervention design and evaluation to ensure that emerging treatments are both equitable and responsive to the lived experiences of marginalized populations.

Finally, implementation and policy-focused research is needed to evaluate the real-world impact of universal trauma screening, integrated care delivery models, and reimbursement structures on maternal mental health outcomes and health equity. Given the substantial health and economic burden associated with untreated perinatal mental health conditions [84, 86], identifying sustainable and cost-effective care models will be critical for informing health system transformation. These efforts will be essential for determining which strategies can be effectively scaled across diverse healthcare settings while improving both maternal and infant outcomes.

## Conclusion

Maternal PTSD is a critical yet underrecognized driver of maternal morbidity, infant health complications, and escalating healthcare costs. Its bidirectional relationship with adverse pregnancy outcomes creates a cycle of trauma and vulnerability that disproportionately affects Black women. Addressing this crisis demands a dual focus: implementing trauma-informed, inclusive care within health systems and advancing research that bridges psychology, public health, and medicine. Culturally responsive interventions, early screening, and structural reforms are essential to reduce maternal mortality, improve infant outcomes, and alleviate the economic burden on healthcare systems. Breaking this cycle is not only a clinical imperative but a moral and public health priority.

## Data Availability

No datasets were generated or analysed during the current study.

## References

[1] Gimbel LA, Weingarten SJ, Smid MC, Hoffman MC. Maternal mental health as a major contributor to maternal mortality. Semin Perinatol. 2024;48(6):151943. 10.1016/j.semperi.2024.151943. 39095259 10.1016/j.semperi.2024.151943

[2] Wisner KL, Murphy C, Thomas MM. Prioritizing maternal mental health in addressing morbidity and mortality. JAMA Psychiatry. 2024;81(5):521. 10.1001/jamapsychiatry.2023.5648. 38381408 10.1001/jamapsychiatry.2023.5648

[3] Hoyert D. Maternal mortality, 2023. 2025. 10.15620/cdc/174577

[4] Maternal mental health leadershimp alliance. Maternal Mental Health State Fact Sheet. 2025.

[5] Centers for disease control and prevention. Pregnancy-Related Deaths: Data from maternal mortality review committees in 36 U.S. states, 2017–2019. Maternal mortality prevention. 2024.

[6] Deshpande NA, Kucirka LM, Smith RN, Oxford CM. Pregnant trauma victims experience nearly 2-fold higher mortality compared to their nonpregnant counterparts. Am J Obstet Gynecol. 2017;217(5):590. e1-590.e9. 10.1016/j.ajog.2017.08.004 28844826

[7] Centers for Disease Control. Four in 5 Pregnancy-Related deaths in the U.S. Are preventable: Data highlight opportunities to better protect Moms. 2022.

[8] Moslimani M, Tamir C, Budiman A, Noe-Bustamante L, Mora L. Facts about the U.S. Black population. 2018.

[9] Giurgescu C, Zenk SN, Templin TN, et al. The impact of neighborhood environment, social support, and avoidance coping on depressive symptoms of pregnant African-American Women. Women’s Health Issues. 2015;25(3):294–302. 10.1016/j.whi.2015.02.001. 25840930 10.1016/j.whi.2015.02.001 PMC4431761

[10] Hawkins M, Mallapareddi A, Misra D. Social mobility and perinatal depression in Black women. Front Health Serv. 2023;3. 10.3389/frhs.2023.1227874. 10.3389/frhs.2023.1227874 PMC10491480 37693235

[11] Farewell CV, Schmiege SJ, Leiferman JA. Racial differences in psychosocial resources and mental and physical health outcomes during pregnancy: a structural equation modeling approach. Matern Health Neonatol Perinatol. 2025;11(1):16. 10.1186/s40748-025-00213-y. 40457470 10.1186/s40748-025-00213-y PMC12131562

[12] Powers A, Woods-Jaeger B, Stevens JS, et al. Trauma, psychiatric disorders, and treatment history among pregnant African American women. Psychol Trauma. 2020;12(2):138–46. 10.1037/tra0000507. 31464464 10.1037/tra0000507 PMC6986992

[13] Floyd James K, Smith BE, Robinson MN, Thomas Tobin CS, Bulles KF, Barkin JL. Factors associated with postpartum maternal functioning in Black Women: A Secondary Analysis. J Clin Med. 2023;12(2):647. 10.3390/jcm12020647. 36675575 10.3390/jcm12020647 PMC9862142

[14] Zivin K, Zhong C, Rodríguez-Putnam A, et al. Suicide mortality during the perinatal period. JAMA Netw Open. 2024;7(6):e2418887. 10.1001/jamanetworkopen.2024.18887. 38935375 10.1001/jamanetworkopen.2024.18887 PMC11211960

[15] Simmons E, Gong J, Daskalopoulou Z, et al. Global contribution of suicide to maternal mortality: a systematic review protocol. BMJ Open. 2024;14(9):e087669. 10.1136/bmjopen-2024-087669. 39284698 10.1136/bmjopen-2024-087669 PMC11409235

[16] Shi P, Ren H, Li H, Dai Q. Maternal depression and suicide at immediate prenatal and early postpartum periods and psychosocial risk factors. Psychiatry Res. 2018;261:298–306. 10.1016/j.psychres.2017.12.085. 29331710 10.1016/j.psychres.2017.12.085

[17] Estriplet T, Morgan I, Davis K, Crear Perry J, Matthews K. Black perinatal mental health: prioritizing maternal mental health to optimize infant health and wellness. Front Psychiatry. 2022;13. 10.3389/fpsyt.2022.807235. 10.3389/fpsyt.2022.807235 PMC9098970 35573337

[18] Kilpatrick SJ. Trauma in pregnancy: an underappreciated cause of maternal death. Am J Obstet Gynecol. 2017;217(5):499–500. 10.1016/j.ajog.2017.09.012. 29110810 10.1016/j.ajog.2017.09.012

[19] Horsch A, Garthus-Niegel S, Ayers S, et al. Childbirth-related posttraumatic stress disorder: definition, risk factors, pathophysiology, diagnosis, prevention, and treatment. Am J Obstet Gynecol. 2024;230(3):S1116–27. 10.1016/j.ajog.2023.09.089. 38233316 10.1016/j.ajog.2023.09.089

[20] Horsch A, Garthus-Niegel S, Ayers S, et al. Childbirth-related posttraumatic stress disorder: definition, risk factors, pathophysiology, diagnosis, prevention, and treatment. Am J Obstet Gynecol. 2024;230(3):S1116–27. 10.1016/j.ajog.2023.09.089. 38233316 10.1016/j.ajog.2023.09.089

[21] Lev-Wiesel R, Chen R, Daphna-Tekoah S, Hod M. Past traumatic events: are they a risk factor for High-risk pregnancy, delivery complications, and postpartum posttraumatic symptoms? J Womens Health. 2009;18(1):119–25. 10.1089/jwh.2008.0774. 10.1089/jwh.2008.0774 19132883

[22] Yonkers KA, Smith MV, Forray A, et al. Pregnant women with posttraumatic stress disorder and risk of preterm birth. JAMA Psychiatry. 2014;71(8):897. 10.1001/jamapsychiatry.2014.558. 24920287 10.1001/jamapsychiatry.2014.558 PMC4134929

[23] Frankham LJ, Thorsteinsson EB, Bartik W. Birth related PTSD and its association with the mother-infant relationship: A meta-analysis. Sex Reproductive Healthc. 2023;38:100920. 10.1016/j.srhc.2023.100920. 10.1016/j.srhc.2023.100920 37847956

[24] Bosquet Enlow M, Egeland B, Carlson E, Blood E, Wright RJ. Mother–infant attachment and the intergenerational transmission of posttraumatic stress disorder. Dev Psychopathol. 2014;26(1):41–65. 10.1017/S0954579413000515. 24059819 10.1017/S0954579413000515 PMC4145695

[25] Sanjuan PM, Fokas K, Tonigan JS, et al. Prenatal maternal posttraumatic stress disorder as a risk factor for adverse birth weight and gestational age outcomes: A systematic review and meta-analysis. J Affect Disord. 2021;295:530–40. 10.1016/j.jad.2021.08.079. 34509068 10.1016/j.jad.2021.08.079 PMC10481878

[26] Upshur CC, Wenz-Gross M, Weinreb L, Moffitt JJA. Using prenatal advocates to implement a psychosocial education intervention for posttraumatic stress disorder during pregnancy: feasibility, care engagement, and predelivery behavioral outcomes. Women’s Health Issues. 2016;26(5):537–45. 10.1016/j.whi.2016.06.003. 27480668 10.1016/j.whi.2016.06.003

[27] Sperlich M, Seng JS, Rowe H, et al. The survivor Moms’ companion: feasibility, safety, and acceptability of a posttraumatic stress specific psychoeducation program for pregnant survivors of childhood maltreatment and sexual trauma. Int J Childbirth. 2011;1(2):122–35. 10.1891/2156-5287.1.2.122.

[28] Valentine SE, Godfrey LB, Gellatly R, et al. Supporting the implementation of written exposure therapy for posttraumatic stress disorder in an obstetrics-substance use disorder clinic in the Northeastern United States. SSM - Mental Health. 2023;4:100256. 10.1016/j.ssmmh.2023.100256. 38645900 10.1016/j.ssmmh.2023.100256 PMC11027481

[29] Nillni YI, Mehralizade A, Mayer L, Milanovic S. Treatment of depression, anxiety, and trauma-related disorders during the perinatal period: A systematic review. Clin Psychol Rev. 2018;66:136–48. 10.1016/j.cpr.2018.06.004. 29935979 10.1016/j.cpr.2018.06.004 PMC6637409

[30] Stevens NR, Miller ML, Soibatian C, et al. Exposure therapy for PTSD during pregnancy: a feasibility, acceptability, and case series study of Narrative Exposure Therapy (NET). BMC Psychol. 2020;8(1):130. 10.1186/s40359-020-00503-4. 33298159 10.1186/s40359-020-00503-4 PMC7727253

[31] Danson L, Ward MJ, Jiang LJ, Miller ML. Posttraumatic stress disorder in the preconception period: an open pilot feasibility study. Matern Health Neonatol Perinatol. 2025;11(1):31. 10.1186/s40748-025-00227-6. 41029782 10.1186/s40748-025-00227-6 PMC12486537

[32] Minhas AS, Ogunwole SM, Vaught AJ, et al. Racial disparities in cardiovascular complications with pregnancy-induced hypertension in the United States. Hypertension. 2021;78(2):480–8. 10.1161/HYPERTENSIONAHA.121.17104. 34098730 10.1161/HYPERTENSIONAHA.121.17104 PMC8266726

[33] Perez Aquine PL. A content analysis of Ethnic minorities in the professional discipline of clinical psychology. Brigham Young University; 2019.

[34] Spates K. The missing link. Sage Open. 2012;2(3). 10.1177/2158244012455179.

[35] Furuta M, Horsch A, Ng ESW, Bick D, Spain D, Sin J. Effectiveness of Trauma-focused psychological therapies for treating post-traumatic stress disorder symptoms in Women following childbirth: A Systematic Review and Meta-analysis. Front Psychiatry. 2018;9. 10.3389/fpsyt.2018.00591. 10.3389/fpsyt.2018.00591 PMC6255986 30515108

[36] Rauch SAM, Eftekhari A, Ruzek JI. Review of exposure therapy: A gold standard for PTSD treatment. J Rehabilitation Res Dev. 2012;49(5):679. 10.1682/JRRD.2011.08.0152. 10.1682/jrrd.2011.08.0152 23015579

[37] Vogel TM, Coffin E. Trauma-informed care on labor and delivery. Anesthesiol Clin. 2021;39(4):779–91. 10.1016/j.anclin.2021.08.007. 34776109 10.1016/j.anclin.2021.08.007

[38] Policy Center for maternal mental health. Black maternal mental health. 2023.

[39] Lewis JA. Contributions of Black psychology scholars to models of racism and health: Applying intersectionality to center Black women. Am Psychol. 2023;78(4):576–88. 10.1037/amp0001141. 37384509 10.1037/amp0001141

[40] Crenshaw K. Demarginalizing the intersection of race and sex: A black feminist critique of antidiscrimination doctrine feminist theory and antiracist politics. University of Chicago Legal Forum. Published online 1989.

[41] Collins P. Black feminist thought. Routledge; 2000.

[42] Howell EA. Reducing disparities in severe maternal morbidity and mortality. Clin Obstet Gynecol. 2018;61(2):387–99. 10.1097/GRF.0000000000000349. 29346121 10.1097/GRF.0000000000000349 PMC5915910

[43] Rosenthal L, Lobel M. Stereotypes of black American Women related to sexuality and motherhood. Psychol Women Q. 2016;40(3):414–27. 10.1177/0361684315627459. 27821904 10.1177/0361684315627459 PMC5096656

[44] van Daalen KR, Kaiser J, Kebede S, et al. Racial discrimination and adverse pregnancy outcomes: a systematic review and meta-analysis. BMJ Glob Health. 2022;7(8):e009227. 10.1136/bmjgh-2022-009227. 35918071 10.1136/bmjgh-2022-009227 PMC9344988

[45] Njoku A, Evans M, Nimo-Sefah L, Bailey J. Listen to the Whispers before they become screams: addressing Black maternal morbidity and mortality in the United States. Healthcare. 2023;11(3):438. 10.3390/healthcare11030438. 36767014 10.3390/healthcare11030438 PMC9914526

[46] OjiNjideka Hemphill N, Crooks N, Zhang W, et al. Obstetric experiences of young black mothers: An intersectional perspective. Soc Sci Med. 2023;317:115604. 10.1016/j.socscimed.2022.115604. 36549014 10.1016/j.socscimed.2022.115604 PMC9854070

[47] Rosenthal L, Lobel M. Gendered racism and the sexual and reproductive health of Black and Latina Women. Ethn Health. 2020;25(3):367–92. 10.1080/13557858.2018.1439896. 29447448 10.1080/13557858.2018.1439896

[48] Powers A, Lipschutz R, McAfee E, et al. Maternal PTSD symptoms and sensitivity during caregiving in early postpartum: The moderating role of resting and reactive RSA in a trauma-exposed sample. Psychol Med. 2025;55:e339. 10.1017/S0033291725102432. 41216728 10.1017/S0033291725102432 PMC12775881

[49] Wosu AC, Gelaye B, Williams MA. Childhood sexual abuse and posttraumatic stress disorder among pregnant and postpartum women: review of the literature. Arch Womens Ment Health. 2015;18(1):61–72. 10.1007/s00737-014-0482-z. 25380784 10.1007/s00737-014-0482-z PMC4308508

[50] Bower JK, Butler BN, Bose-Brill S, Kue J, Wassel CL. Racial/Ethnic differences in diabetes screening and hyperglycemia among US Women after gestational diabetes. Prev Chronic Dis. 2019;16:190144. 10.5888/pcd16.190144. 10.5888/pcd16.190144 PMC6824147 31651379

[51] Lacey KK, Mouzon DM, Parnell RN, Laws T. Severe intimate partner violence, sources of stress and the Mental Health of U.S. Black Women. J Womens Health. 2021;30(1):17–28. 10.1089/jwh.2019.8215. 10.1089/jwh.2019.8215 32813617

[52] Koch AR, Geller SE. Addressing maternal deaths due to violence: the Illinois experience. Am J Obstet Gynecol. 2017;217(5):556. 10.1016/j.ajog.2017.08.005. 10.1016/j.ajog.2017.08.005 28844823

[53] Wiens T. When Men Murder Women: An Analysis of 2022 Homicide Data. 2024.

[54] Al-abri K, Edge D, Armitage CJ. Correction to: Prevalence and correlates of perinatal depression. Soc Psychiatry Psychiatr Epidemiol. 2023;58(11):1591–1591. 10.1007/s00127-023-02468-2. 37079047 10.1007/s00127-023-02468-2 PMC10562261

[55] Jones AL, Rafferty J, Cochran SD, Abelson J, Hanna MR, Mays VM, Prevalence. Severity and burden of post-traumatic stress disorder in Black Men and Women across the adult life span. J Aging Health. 2022;34(3):401–12. 10.1177/08982643221086071. 35510479 10.1177/08982643221086071 PMC9175561

[56] Kapaya M, Boulet SL, Warner L, Harrison L, Fowler D. Intimate partner violence before and during pregnancy, and prenatal counseling among Women with a recent live birth, United States, 2009–2015. J Womens Health. 2019;28(11):1476–86. 10.1089/jwh.2018.7545. 10.1089/jwh.2018.7545 PMC10936761 31460827

[57] Nguyen TT, Criss S, Kim M, et al. Racism during pregnancy and birthing: experiences from Asian and pacific islander, Black, Latina, and Middle Eastern Women. J Racial Ethn Health Disparities. 2023;10(6):3007–17. 10.1007/s40615-022-01475-4. 36449130 10.1007/s40615-022-01475-4 PMC9713108

[58] Spurlock EJ, Pickler RH. Birth experience among Black Women in the United States: A qualitative Meta-synthesis. J Midwifery Womens Health. 2024;69(5):697–717. 10.1111/jmwh.13628. 38561916 10.1111/jmwh.13628

[59] Siddiqi M, Guiab K, Roberts A, et al. Maternal outcomes after Trauma in pregnancy: A national database study. Am Surg. 2022;88(8):1760–5. 10.1177/00031348221083940. 35333642 10.1177/00031348221083940

[60] DuMonthier A, Childers C, Milli J. The status of Black women in the United States: Executive summary. 2020.

[61] Kang-Yi CD, Kornfield SL, Epperson CN, Mandell DS. Relationship between pregnancy complications and psychiatric disorders: A population-based study with a matched control group. 2018;69(3):300–7. 10.1176/appi.ps.201700097 10.1176/appi.ps.201700097 PMC5937933 29137553

[62] Barlow JN. Uncovering the Trauma pregnant black Women experience in the U.S. 2020.

[63] Osborne AD, Yasova Barbeau D, Gladdis T, et al. Understanding and addressing mental health challenges of families admitted to the neonatal intensive care unit. J Perinatol. 2025;45(6):873–80. 10.1038/s41372-024-02187-9. 39643695 10.1038/s41372-024-02187-9 PMC12144238

[64] Bauman BL, Ko JY, Cox S, et al. Vital Signs: Postpartum depressive symptoms and provider discussions about perinatal depression — United States, 2018. MMWR Morb Mortal Wkly Rep. 2020;69(19):575–81. 10.15585/mmwr.mm6919a2. 32407302 10.15585/mmwr.mm6919a2 PMC7238954

[65] Meaney MJ, Szyf M, Seckl JR. Epigenetic mechanisms of perinatal programming of hypothalamic-pituitary-adrenal function and health. Trends Mol Med. 2007;13(7):269–77. 10.1016/j.molmed.2007.05.003. 17544850 10.1016/j.molmed.2007.05.003

[66] Wu Y, De Asis-Cruz J, Limperopoulos C. Brain structural and functional outcomes in the offspring of women experiencing psychological distress during pregnancy. Mol Psychiatry. 2024;29(7):2223–40. 10.1038/s41380-024-02449-0. 38418579 10.1038/s41380-024-02449-0 PMC11408260

[67] Jones K, Acevedo BP, Avalos LA, et al. Association between maternal perceived stress during pregnancy and offspring DNA methylation changes in HPA axis genes at birth in the ECHO Consortium. Environ Epigenet. 2025;11(1). 10.1093/eep/dvaf024. 10.1093/eep/dvaf024 PMC12462613 41020071

[68] Dieckmann L, Czamara D. Epigenetics of prenatal stress in humans: the current research landscape. Clin Epigenetics. 2024;16(1):20. 10.1186/s13148-024-01635-9. 38308342 10.1186/s13148-024-01635-9 PMC10837967

[69] Minhas AS, Ying W, Ogunwole SM, et al. The association of adverse pregnancy outcomes and cardiovascular disease: Current knowledge and future directions. Curr Treat Options Cardiovasc Med. 2020;22(12):61. 10.1007/s11936-020-00862-6. 35296064 10.1007/s11936-020-00862-6 PMC8923621

[70] Van den Bergh BRH, van den Heuvel MI, Lahti M, et al. Prenatal developmental origins of behavior and mental health: The influence of maternal stress in pregnancy. Neurosci Biobehav Rev. 2020;117:26–64. 10.1016/j.neubiorev.2017.07.003. 28757456 10.1016/j.neubiorev.2017.07.003

[71] Crear-Perry J, Correa-de-Araujo R, Lewis Johnson T, McLemore MR, Neilson E, Wallace M. Social and structural determinants of health inequities in Maternal Health. J Womens Health. 2021;30(2):230–5. 10.1089/jwh.2020.8882. 10.1089/jwh.2020.8882 PMC8020519 33181043

[72] Sriram K, Umer A, Hamilton C, John CC. Abstract P387: Risk factors for neonatal intensive care unit admission; Project Watch, 2012–2017. Circulation. 2020;141(Suppl1). 10.1161/circ.141.suppl_1.P387.

[73] Alhamawi NJ, Alharbi HA, Alqahtani MH. Reasons and factors affecting the neonatal intensive care Unit (NICU) Length of Stay of Full-Term Newborns: A Systematic Review. Cureus. Published online November 18, 2024. 10.7759/cureus.73892 10.7759/cureus.73892 PMC11655050 39697931

[74] Devita S, Bozicevic L, Deforges C, et al. Early mother-infant interactions within the context of childbirth-related posttraumatic stress symptoms. J Affect Disord. 2024;365:24–31. 10.1016/j.jad.2024.08.025. 39151764 10.1016/j.jad.2024.08.025

[75] Alsallum FS, Boyle B, McLaughlin D, McGowan I. The risk factors of post-traumatic stress disorder among parents of neonatal intensive care unit infants: A systematic review. J Neonatal Nurs. 2025;31(2):101620. 10.1016/j.jnn.2025.101620.

[76] Huffhines L, Coe JL, Busuito A, Seifer R, Parade SH. Understanding links between maternal perinatal posttraumatic stress symptoms and infant socioemotional and physical health. Infant Ment Health J. 2022;43(3):474–92. 10.1002/imhj.21985. 35513001 10.1002/imhj.21985 PMC9177799

[77] Bosquet Enlow M, Kitts RL, Blood E, Bizarro A, Hofmeister M, Wright RJ. Maternal posttraumatic stress symptoms and infant emotional reactivity and emotion regulation. Infant Behav Dev. 2011;34(4):487–503. 10.1016/j.infbeh.2011.07.007. 21862136 10.1016/j.infbeh.2011.07.007 PMC3180882

[78] Chasson M, Khoury J, Bosquet Enlow M, Lyons-Ruth K. Maternal caregiving moderates relations between maternal childhood maltreatment and infant cortisol regulation. J Child Psychol Psychiatry. 2025;66(11):1627–41. 10.1111/jcpp.14171. 40197551 10.1111/jcpp.14171 PMC12571947

[79] Kjerulff KH, Attanasio LB, Sznajder KK, Brubaker LH. A prospective cohort study of post-traumatic stress disorder and maternal-infant bonding after first childbirth. J Psychosom Res. 2021;144:110424. 10.1016/j.jpsychores.2021.110424. 33756149 10.1016/j.jpsychores.2021.110424 PMC8101703

[80] Martin RCB, Bridgett DJ, Mayes LC, Rutherford HJV. Maternal working memory, emotion regulation, and responsivity to infant distress. J Appl Dev Psychol. 2020;71:101202. 10.1016/j.appdev.2020.101202.

[81] Bozicevic L, De Pascalis L, Cooper P, Murray L. The role of maternal sensitivity, infant temperament, and emotional context in the development of emotion regulation. Sci Rep. 2025;15(1):17271. 10.1038/s41598-025-01714-8. 40389444 10.1038/s41598-025-01714-8 PMC12089384

[82] Devita S, Deforges C, Bickle-Graz M, Tolsa JF, Sandoz V, Horsch A. Maternal childbirth-related posttraumatic stress symptoms, bonding, and infant development: a prospective study. J Reprod Infant Psychol. 2025;43(3):718–32. 10.1080/02646838.2023.2261057. 37740725 10.1080/02646838.2023.2261057

[83] Brown CC, Adams CE, George KE, Moore JE. Mental health conditions increase severe maternal morbidity By 50% and Cost $102 Million yearly in the United States. Health Aff. 2021;40(10):1575–84. 10.1377/hlthaff.2021.00759. 10.1377/hlthaff.2021.00759 PMC8759410 34606352

[84] Valencia Z, Bozzi D, Sen A, Martin K. The price of childbirth in the U.S. Tops $13,000 in 2020. 2022.

[85] Fox B. Pregnancy and delivery complications cost the United States billions in health care expenses, lost productivity, and social support services. 2021.

[86] Hall SV, Bell S, Courant A, Admon LK, Zivin K. Perinatal posttraumatic stress disorder diagnoses among commercially insured people increased, 2008–20. Health Aff. 2024;43(4):504–13. 10.1377/hlthaff.2023.01447. 10.1377/hlthaff.2023.01447 PMC11225106 38560801

[87] Khoramroudi R. The prevalence of posttraumatic stress disorder during pregnancy and postpartum period. J Family Med Prim Care. 2018;7(1):220–3. 10.4103/jfmpc.jfmpc_272_17. 29915763 10.4103/jfmpc.jfmpc_272_17 PMC5958573

[88] Winger A, Rae M, Cox C. Health costs associated with pregnancy, childbirth, and infant care. 2025.

[89] Martin J, Osterman M. Increases in neonatal intensive care admissions in the United States, 2016–2023. 2025. 10.15620/cdc/174581 10.15620/cdc/174581 PMC12038910 40168594

[90] Wilson CA, Bublitz M, Chandra P, et al. A global perspective: Access to mental health care for perinatal populations. Semin Perinatol. 2024;48(6):151942. 10.1016/j.semperi.2024.151942. 39048414 10.1016/j.semperi.2024.151942

[91] Reist C, Petiwala I, Latimer J, et al. Collaborative mental health care: A narrative review. Medicine. 2022;101(52):e32554. 10.1097/MD.0000000000032554. 36595989 10.1097/MD.0000000000032554 PMC9803502

[92] Waqas A, Koukab A, Meraj H, et al. Screening programs for common maternal mental health disorders among perinatal women: report of the systematic review of evidence. BMC Psychiatry. 2022;22(1):54. 10.1186/s12888-022-03694-9. 35073867 10.1186/s12888-022-03694-9 PMC8787899

[93] Rauch SAM, Eftekhari A, Ruzek JI. Review of exposure therapy: A gold standard for PTSD treatment. J Rehabilitation Res Dev. 2012;49(5):679. 10.1682/JRRD.2011.08.0152. 10.1682/jrrd.2011.08.0152 23015579

[94] Furuta M, Horsch A, Ng ESW, Bick D, Spain D, Sin J. Effectiveness of Trauma-focused psychological therapies for treating post-traumatic stress disorder symptoms in Women following childbirth: a Systematic review and meta-analysis. Front Psychiatry. 2018;9. 10.3389/fpsyt.2018.00591. 10.3389/fpsyt.2018.00591 PMC6255986 30515108

[95] Miller ML, Wasson RS, Jiang LJ, Ward MJ, Meyer DJ, Haas DM. Narrative exposure therapy: expanding virtual treatment of posttraumatic stress disorder to the postpartum period. J Aggress Maltreat Trauma. 2025;34(4):538–57. 10.1080/10926771.2025.2491772. 40395722 10.1080/10926771.2025.2491772 PMC12088625

[96] Neal-Barnett A, Stadulis RE, Ayoade EE, McGhee-Dinvaut A. A pilot study exploring the feasibility of virtual written exposure therapy with underserved Black Perinatal Women. J Racial Ethn Health disparities published online Oct. 2024;14. 10.1007/s40615-024-02203-w. 10.1007/s40615-024-02203-w PMC12644141 39400625

[97] Valentine SE, Godfrey LB, Gellatly R, et al. Supporting the implementation of written exposure therapy for posttraumatic stress disorder in an obstetrics-substance use disorder clinic in the Northeastern United States. SSM - Mental Health. 2023;4:100256. 10.1016/j.ssmmh.2023.100256. 38645900 10.1016/j.ssmmh.2023.100256 PMC11027481

[98] Nillni YI, Baul TD, Paul E, Godfrey LB, Sloan DM, Valentine SE. Written exposure therapy for treatment of perinatal PTSD among women with comorbid PTSD and SUD: A pilot study examining feasibility, acceptability, and preliminary effectiveness. Gen Hosp Psychiatry. 2023;83:66–74. 10.1016/j.genhosppsych.2023.04.013. 37119780 10.1016/j.genhosppsych.2023.04.013 PMC10587907

[99] Meltzer-Brody S, Brandon AR, Pearson B, et al. Evaluating the clinical effectiveness of a specialized perinatal psychiatry inpatient unit. Arch Womens Ment Health. 2014;17(2):107–13. 10.1007/s00737-013-0390-7. 24201978 10.1007/s00737-013-0390-7 PMC3961543

[100] Battle CL, Howard MM. A mother–baby psychiatric day hospital: History, rationale, and why perinatal mental health is important for obstetric medicine. Obstet Med. 2014;7(2):66–70. 10.1177/1753495X13514402. 27512426 10.1177/1753495X13514402 PMC4934950

[101] Posmontier B, Geller PA, Horowitz JA, Elgohail M, Chiarello L. Intensive perinatal mental health programs in the United States: A Call to Action. 2022;73(8):930–2. 10.1176/appi.ps.202100384 10.1176/appi.ps.202100384 35080417

[102] Tadanki D, Kaza PS, Meisinger E, et al. Comprehensive review of the impact of maternal stress on fetal development. Pediatr Discovery. 2025;3(3). 10.1002/pdi3.70004. 10.1002/pdi3.70004 PMC12483304 41036306

